# Supplementary material for: WU-CRISPR: characteristics of functional guide RNAs for the CRISPR/Cas9 system
Source: Genome Biol. 2015 Nov 2;16:218. doi: 10.1186/s13059-015-0784-0 (PMC4629399; doi:10.1186/s13059-015-0784-0)
Supplement: Additional file 1: — Supplementary Tables S1–S3. (PDF 285 kb) [file 13059_2015_784_MOESM1_ESM.pdf]

Table S1. Significant trinucleoside counts in the guide sequence of functional sgRNAs*

Trinucleoside Count	Enrichment Ratio	P-Value
GGG	0.39	4.9E-13
GGC	0.51	3.1E-10
GUA	2.83	9.0E-08
AGA	2.39	2.9E-07
UAG	2.69	9.3E-06
GCC	0.63	9.7E-06
UUU	0.40	4.1E-05
UAC	2.09	5.5E-05
ACC	1.72	7.9E-05
AAG	1.96	8.0E-05
AUU	0.48	2.0E-04
AUA	2.94	2.6E-04
ACA	1.68	2.7E-04
CAA	1.75	3.4E-04
CCU	0.65	3.5E-04
CGG	0.54	3.6E-04
CCA	1.42	5.0E-04
AGU	1.62	1.1E-03
AUG	1.63	1.2E-03
UUG	0.61	1.7E-03
CGU	1.75	4.5E-03
GCG	0.61	6.0E-03
GAC	1.44	6.3E-03
UGG	0.76	6.9E-03
CUU	0.68	7.0E-03
UCA	1.42	9.4E-03
CAG	1.26	1.5E-02
GGU	0.73	1.7E-02
CUA	1.70	1.9E-02
UCU	0.72	2.6E-02
CAC	1.34	2.7E-02
UUC	0.76	3.3E-02
GCA	1.30	3.4E-02
GUU	0.73	3.8E-02
CCC	0.76	4.1E-02
GAU	1.38	4.6E-02
ACG	1.44	4.8E-02

* The enrichment ratio was determined by comparing the average nucleoside counts in the guide sequence of functional sgRNAs to that of non-functional sgRNAs. The p-values were calculated with Student's t-test.

Table S2. Base positions in the target site that were significantly associated with sgRNA activities*

Position-Specific Nucleoside	Enrichment Ratio	P-Value
3:A	1.81	3.9E-05
3:C	0.59	6.2E-06
3:G	0.78	5.2E-02
3:T	1.49	3.8E-03
9:A	1.62	8.7E-04
9:C	0.97	8.1E-01
9:G	0.71	6.0E-03
9:T	0.99	9.3E-01
10:A	1.47	1.6E-02
10:C	1.12	3.3E-01
10:G	0.76	8.0E-03
10:T	0.96	7.8E-01
11:A	1.96	1.0E-05
11:C	0.74	9.6E-03
11:G	0.93	5.2E-01
11:T	0.89	4.6E-01
12:A	1.73	2.0E-04
12:C	0.71	4.2E-03
12:G	1.04	7.4E-01
12:T	0.89	3.9E-01
14:A	1.63	4.2E-04
14:C	0.86	2.1E-01
14:G	0.61	8.9E-06
14:T	1.55	3.9E-03
15:A	1.66	2.9E-04
15:C	0.78	6.5E-02
15:G	0.77	1.4E-02
15:T	1.14	3.4E-01
16:A	1.58	4.9E-03
16:C	1.91	2.9E-08
16:G	0.52	4.8E-10
16:T	0.77	8.7E-02
17:A	1.62	1.9E-03
17:C	1.41	3.4E-03
17:G	0.66	2.0E-04
17:T	0.75	4.5E-02
18:A	1.51	5.8E-03
18:C	1.36	3.7E-03
18:G	0.81	1.1E-01
18:T	0.54	2.0E-05
19:A	1.67	2.2E-03
19:C	0.97	7.6E-01
19:G	1.53	6.4E-04
19:T	0.39	7.2E-10

20:A	2.23	4.6E-07
20:C	0.35	4.9E-19
20:G	3.16	9.5E-19
20:T	0.51	7.0E-06
21:A	0.98	8.6E-01
21:C	1.64	5.4E-03
21:G	1.29	3.7E-02
21:T	0.66	9.3E-05
24:A	1.38	1.3E-02
24:C	1.30	4.1E-02
24:G	0.41	4.7E-13
24:T	1.59	1.2E-03

* The PAM domain (NGG) occupies positions 21-23.

Table S3. Significant features used to predict sgRNA potency.

Feature Name	Enrichment Ratio	P-Value	Feature Type	In Doench study
Accessibility position 15	1.23	1.8E-03	Binary (0 or 1)	No
Accessibility position 18	1.37	8.9E-07	Binary (0 or 1)	No
Accessibility position 19	1.88	3.2E-13	Binary (0 or 1)	No
Accessibility position 20	1.78	3.0E-10	Binary (0 or 1)	No
Accessibility position 21	0.94	1.9E-03	Binary (0 or 1)	No
Accessibility position 51	1.57	1.1E-03	Binary (0 or 1)	No
Accessibility position 52	1.61	3.0E-05	Binary (0 or 1)	No
Accessibility position 53	1.17	4.8E-04	Binary (0 or 1)	No
Count A	1.39	9.3E-18	Numerical	No
Count G	0.92	6.2E-03	Numerical	No
Count U	0.89	1.9E-03	Numerical	No
Count AC	1.47	1.2E-08	Numerical	No
Count AG	1.43	1.3E-09	Numerical	No
Count CA	1.38	6.7E-09	Numerical	No
Count CU	0.82	1.2E-03	Numerical	No
Count GA	1.37	4.2E-06	Numerical	No
Count GC	0.77	3.2E-06	Numerical	No
Count GG	0.64	2.3E-11	Numerical	No
Count UA	1.84	1.1E-07	Numerical	No
Count UU	0.59	7.5E-08	Numerical	No
Count AAG	1.96	8.0E-05	Numerical	No
Count ACA	1.68	2.7E-04	Numerical	No
Count ACC	1.72	7.9E-05	Numerical	No
Count AGA	2.39	2.9E-07	Numerical	No
Count AGU	1.62	1.1E-03	Numerical	No
Count AUA	2.94	2.6E-04	Numerical	No
Count AUG	1.63	1.2E-03	Numerical	No
Count AUU	0.48	2.0E-04	Numerical	No
Count CAA	1.75	3.4E-04	Numerical	No
Count CCA	1.42	5.0E-04	Numerical	No
Count CCU	0.65	3.5E-04	Numerical	No
Count CGG	0.54	3.6E-04	Numerical	No
Count CGU	1.75	4.5E-03	Numerical	No
Count CUU	0.68	7.0E-03	Numerical	No
Count GAC	1.44	6.3E-03	Numerical	No
Count GCC	0.63	9.7E-06	Numerical	No
Count GCG	0.61	6.0E-03	Numerical	No
Count GGC	0.51	3.1E-10	Numerical	No
Count GGG	0.39	4.9E-13	Numerical	No
Count GUA	2.83	9.0E-08	Numerical	No
Count UAC	2.09	5.5E-05	Numerical	No
Count UAG	2.69	9.3E-06	Numerical	No
Count UCA	1.42	9.4E-03	Numerical	No
Count UGG	0.76	6.9E-03	Numerical	No

Count UUG	0.61	1.7E-03	Numerical	No
Count UUU	0.40	4.1E-05	Numerical	No
Duplex stability gRNA	0.93	1.5E-08	Numerical	No
Duplex stability seed	0.91	4.9E-10	Numerical	No
Folding stability gRNA	0.61	6.7E-11	Numerical	No
GC Content	0.93	2.1E-05	Numerical	Yes
Position 3 A	1.81	3.9E-05	Binary (0 or 1)	No
Position 3 C	0.59	6.2E-06	Binary (0 or 1)	Yes
Position 3 U	1.49	3.8E-03	Binary (0 or 1)	No
Position 9 A	1.62	8.7E-04	Binary (0 or 1)	No
Position 9 G	0.71	6.0E-03	Binary (0 or 1)	No
Position 10 G	0.76	8.0E-03	Binary (0 or 1)	No
Position 11 A	1.96	1.0E-05	Binary (0 or 1)	Yes
Position 11 C	0.74	9.6E-03	Binary (0 or 1)	Yes
Position 12 A	1.73	2.0E-04	Binary (0 or 1)	Yes
Position 12 C	0.71	4.2E-03	Binary (0 or 1)	Yes
Position 14 A	1.63	4.2E-04	Binary (0 or 1)	No
Position 14 G	0.61	8.9E-06	Binary (0 or 1)	Yes
Position 14 U	1.55	3.9E-03	Binary (0 or 1)	No
Position 15 A	1.66	2.9E-04	Binary (0 or 1)	Yes
Position 16 A	1.58	4.9E-03	Binary (0 or 1)	No
Position 16 C	1.91	2.9E-08	Binary (0 or 1)	No
Position 16 G	0.52	4.8E-10	Binary (0 or 1)	Yes
Position 17 A	1.62	1.9E-03	Binary (0 or 1)	No
Position 17 C	1.41	3.4E-03	Binary (0 or 1)	No
Position 17 G	0.66	2.0E-04	Binary (0 or 1)	Yes
Position 18 A	1.51	5.8E-03	Binary (0 or 1)	No
Position 18 C	1.36	3.7E-03	Binary (0 or 1)	Yes
Position 18 U	0.54	2.0E-05	Binary (0 or 1)	Yes
Position 19 A	1.67	2.2E-03	Binary (0 or 1)	No
Position 19 G	1.53	6.4E-04	Binary (0 or 1)	No
Position 19 U	0.39	7.2E-10	Binary (0 or 1)	Yes
Position 20 A	2.23	4.6E-07	Binary (0 or 1)	No
Position 20 C	0.35	4.9E-19	Binary (0 or 1)	Yes
Position 20 G	3.16	9.5E-19	Binary (0 or 1)	Yes
Position 20 U	0.51	7.0E-06	Binary (0 or 1)	Yes
Position 21 C	1.64	5.4E-03	Binary (0 or 1)	Yes
Position 21 U	0.66	9.3E-05	Binary (0 or 1)	Yes
Position 24 G	0.41	4.7E-13	Binary (0 or 1)	Yes
Position 24 U	1.59	1.2E-03	Binary (0 or 1)	Yes
Repetitive bases	0.24	1.3E-11	Binary (0 or 1)	No
UUU in the seed region	0.10	8.8E-07	Binary (0 or 1)	No
